# The IGF-1R Inhibitor NVP-AEW541 Causes Insulin-Independent and Reversible Cardiac Contractile Dysfunction

**DOI:** 10.3390/biomedicines10082022

**Published:** 2022-08-19

**Authors:** Christina Schenkl, Andrea Schrepper, Estelle Heyne, Torsten Doenst, Michael Schwarzer

**Affiliations:** Department of Cardiothoracic Surgery, University Hospital Jena, Friedrich Schiller University of Jena, Am Klinikum 1, 07747 Jena, Germany

**Keywords:** NVP-AEW541, small molecule, cardiac function, cardiac substrate oxidation, insulin-like growth factor 1, insulin resistance, cancer treatment

## Abstract

The antitumor treatment NVP-AEW541 blocks IGF-1R. IGF-1R signaling is crucial for cardiac function, but the cardiac effects of NVP-AEW541 are ill defined. We assessed NVP-AEW541′s effects on cardiac function and insulin response in vivo and in isolated working hearts. We performed a dose–response analysis of NVP-AEW541 in male, 3-week-old rats and assessed the chronic effects of the clinically relevant dose in adult rats. We performed glucose tolerance tests and echocardiography; assessed the expression and phosphorylation of InsR/IGF-1R and Akt in vivo; and measured substrate oxidation, contractile function, and insulin response in the isolated working hearts. NVP-AEW541 caused dose-dependent growth retardation and impaired glucose tolerance in the juvenile rats. In the adults, NVP-AEW541 caused a continuously worsening depression of cardiac contractility, which recovered within 2 weeks after cessation. Cardiac Akt protein and phosphorylation were unchanged and associated with InsR upregulation. An acute application of NVP-AEW541 in the working hearts did not affect cardiac power but eliminated insulin’s effects on glucose and fatty acid oxidation. The systemic administration of NVP-AEW541 caused dose- and time-dependent impairment of glucose tolerance, growth, and cardiac function. Because cardiac insulin signaling was maintained in vivo but absent in vitro and because contractile function was not affected in vitro, a direct link between insulin resistance and contractile dysfunction appears unlikely.

## 1. Introduction

Insulin-like growth factor 1 (IGF-1) promotes the proliferation, differentiation, and survival of cells and tissues but also those of various IGF-1-sensitive tumor types, such as breast cancer, rhabdoid tumors, and bone and soft tissue sarcomas. Therefore, IGF-1 and its receptor, IGF-1R, have become targets of a new promising strategy to treat such tumors. IGF-1 binds to IGF-1R and, to a lesser extent, also to the structurally very similar insulin receptor. Such binding leads to the auto-phosphorylation and activation of the receptors. Once activated, the receptors initiate a cascade of downstream signals involving the protein kinase B (Akt). Akt is a key protein in both signaling cascades, resulting in anabolic cellular conditions and the initiation of tissue development and growth [1]. Recently, synthetic, small-molecule tyrosine kinase inhibitors aroused attention for the treatment of tumors. These compounds are structurally very similar to IGF-1 and bind competitively to IGF-1R, suppressing its auto-phosphorylation to silence its signaling and, thereby, preventing the growth and progression of tumor tissue.

One representative of small-molecule IGF-1R inhibitors is NVP-AEW541, a pyrrolo [2,3-*d*]pyrimidine derivative small-molecular-weight tyrosine kinase inhibitor. NVP-AEW541 is described as being very specific for IGF-1R in cells, having a much higher affinity (up to 30-fold) for IGF-1R than for structurally homologue tyrosine kinases, e.g., the insulin receptor [2,3]. Its antitumor activity has been proven in vitro and in vivo and shows promising therapeutic potential [2,4].

Similar to all tissues and cell types, the heart expresses IGF-1R, which is involved in cardiac development and the maintenance of cellular homeostasis [5,6]. IGF-1 promotes cardiac growth, contractility, stroke volume, and ejection fraction [7,8,9]. Thus far, cell culture and animal studies have mainly focused on treatment effects in target (tumor) tissues. Preliminary clinical trials with NVP-AEW541 and similar small molecules assessed systemic parameters and basal cardiac contractile function. Available data sets from clinical trials demonstrate the occurrence of hyperglycemia, cardiac arrhythmia, and tachycardia (clinicaltrials.gov). However, the specific impact of NVP-AEW541 application on cardiac function and metabolism in vivo or in vitro has not been assessed to date.

We assessed the cardiac effects of systemic NVP-AEW541 application on contractile function and insulin response in rats in vivo, and we also evaluated its influence on the cardiac substrate metabolism and insulin response in isolated working hearts.

## 2. Materials and Methods

### 2.1. Experimental Design/Study Protocol

All animal studies were approved by the responsible animal welfare committee (Thüringer Landesamt für Verbraucherschutz) and registered as 22-2684-04-02-002. Male Sprague Dawley rats were randomly assigned to a treatment group or the control group. In the first set, three-week-old juvenile rats were given the IGF-1R inhibitor NVP-AEW541 with drinking water. We used daily doses of 80, 160, and 240 mg/kg dissolved in 25 mM L(+)-tartaric acid as solvent. The control group received 25 mM tartaric acid only.

**Figure 1:**
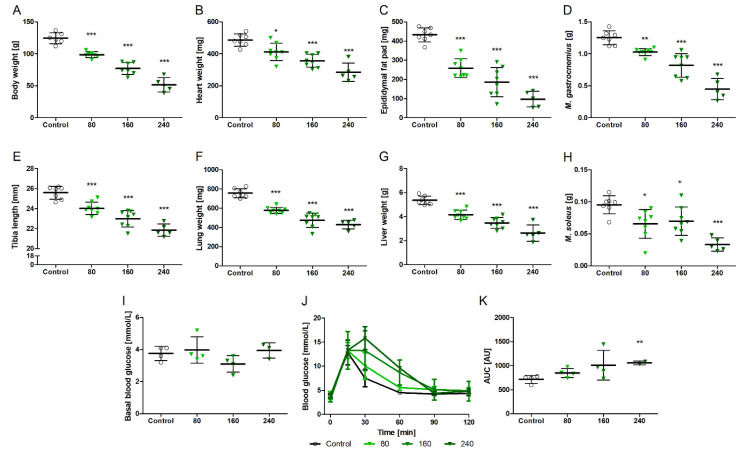
Effects of two weeks of NVP-AEW541 application (80, 160, 240 mg/kg) on the body and organ weights and the glucose tolerance of juvenile rats. Body weight (**A**), organ weights (**B**–**H**) and tibia length, fasting blood glucose (**I**), time course of glucose removal (**J**), and glucose tolerance (**K**) as determined by area under the curve (AUC). Data are presented as mean ± SD, *n* = 5–8 per group. Significant differences * *p* < 0.05; ** *p* < 0.01; *** *p* < 0.001 vs. vehicle.

The lowest dose of 80 mg/kg BW led to disturbed insulin sensitivity in the juvenile rats (Figure 1). Hence, for ongoing experiments, the adult rats were administered this clinically relevant dose of NVP-AEW541. Treatments were planned for 4 weeks, with a midterm analysis of glucose tolerance, morphometric data, and protein expressions after 2 weeks. However, treatment had to be terminated after 3 weeks due to the deterioration of the health of the animals. Therefore, post-mortem results were obtained after 14 days of treatment, while echocardiographic in vivo analyses were extended up to three weeks.

### 2.2. Glucose Tolerance Test

Glucose tolerance tests were performed after one week of NVP-AW541 treatment to allow for the sufficient wash-out and recovery of the animals. Prior to the glucose tolerance tests, animals were fasted for 6 h. Fasting blood glucose was measured in capillary blood with a commercially available glucometer (FreeStyle) prior to the test and documented. For the glucose tolerance tests, rats were injected with glucose at doses of 2 g/kg i.p. Blood glucose levels were measured repeatedly after 15, 30, 60, 120, and 180 min and plotted graphically. The area under the curve (AUC) was calculated as integral in arbitrary units (AU).

### 2.3. Cardiac Function In Vivo

Echocardiography was performed prior to NVP-AEW541 treatment and weekly for five weeks. Echocardiographic examination was performed as previously described by us [10]. The animals were anesthetized with 5% isoflurane. The animals’ chests were shaved, and the rats were examined in supine position with a Vevo770 and a 17.5 Mhz RMV716 array transducer (VisualSonics^®^). Two-dimensional short-axis views of the left ventricle at the papillary muscle level were obtained. Two-dimensional-guided M-mode tracings were recorded with a sweep speed of 100 mm/s. The following parameters were obtained: ejection fraction (EF), left ventricular posterior wall thickness in diastole and systole, and end-diastolic dimensions (LVEDD).

The mitral inflow pattern was assessed from the apical 4-chamber view with the use of pulsed-wave Doppler and the sample volume placed at the mitral leaflet tips. Mitral inflow Doppler measurements included early (E) ventricular filling velocity and the deceleration time (DT) of the E-wave. The derived parameters of the mitral inflow Doppler involved E/DT. All values were averaged from 3 consecutive measurements.

### 2.4. Isolated Working Rat Heart Perfusion

We have previously described the technique of isolated working heart perfusion in detail [11]. In the present study, the perfusate contained either 10 µM NVP-AEW541 dissolved in 25 mM tartaric acid or tartaric acid only. The NVP-AEW541 concentration for isolated working heart perfusion was based on plasma levels resulting from comparable administered dosages measured elsewhere [12]. NVP-AEW541 and tartaric acid were added in a total volume of 100 µL/2 L perfusate. In the presence of either NVP-AEW541 or tartaric acid, hearts were perfused with both glucose (5 mmol/L) and oleate (0.4 mmol/L) as substrates. After 30 min, insulin (0.5 mU/mL) was added, and the hearts were perfused for further 30 min. Cardiac power (*CP*) was assessed every 5 min, and its changes were calculated as follows:

$$CP\;\left[\mathrm{mW}\right]=\frac{\left(\sum^{\;}cardiac\;output\;\left[\frac{\mathrm{mL}}{\min}\right]\right)\times 100\;\mathrm{cm}}{612}$$

Every 5 min, a sample of coronary effluent was withdrawn for the measurement of fatty acid (FAO) and glucose oxidation (GO), determined as the production of ^3^H_2_O from [9,10-3H]oleate and of ^14^CO_2_ from [U-14C]glucose (both from Perkin-Elmer), respectively. The methods for isolated working hearts have been previously described in detail [11]. Briefly, for the estimation of glucose oxidation, exhausted ^14^CO_2_ and the ^14^CO_2_ content of the perfusate were captured in 0.33 M benzethonium hydroxide. For the estimation of fatty acid oxidation, perfusate samples containing the metabolic end product of fatty acids, ^3^H_2_O, were separated on a selective resin. Samples containing ^14^CO_2_ or ^3^H_2_O were incubated with UltimaGold, and radioactivity was measured in a scintillation counter.

### 2.5. Protein Expression and Insulin Signaling Cascade

Rats were euthanized using thiopental (300 mg/kg). Morphometric data were collected during organ harvesting. For the characterization of the insulin-dependent phosphorylation of InsR/IGF-1R, half of the NVP-AEW541-treated animals were randomly selected and stimulated with insulin (1 U/kg BW). Insulin was injected in the V. portae, and organs were harvested 5 min later. Cardiac tissue was snap-frozen and stored at −80 °C for a further analysis of protein expression. For a Western blot analysis, the protein concentration of homogenized heart tissue (ventricles) was measured according to the Bradford method. Cardiac protein (approx. 40 μg) was loaded to a 10% polyacrylamide gel and separated at 40 mA for 1 h. After SDS-PAGE, the proteins were transferred to a PDVF membrane and incubated with primary antibodies against InsR/IGF-1R (Tyr999) (#3025, Cell Signaling), Phospho-InsR/IGF-1R (Tyr 1135/1136 and Tyr1150/1151) (#3024S, Cell Signaling), Akt (binding to peptide corresponding to the carboxy-terminal sequence) (#9272S, Cell Signaling), Phospho-Akt (Ser473) (#9271S, Cell Signaling), and secondary antibody (#NA934V, AbChem). Original gels are shown in Supplemental Material. Bands were visualized with chemiluminescence solution (Serva) and 1% H_2_O_2_ using an LAS4000 imaging system (GE Healthcare). Respective bands were semi-quantified with ImageJ software in a blinded manner.

### 2.6. Statistical Analyses

Data are presented as mean ± standard deviation (SD). Data were tested for normal distribution, applying the Kolmogorov–Smirnov Test. Parametric data were analyzed using a one-way analysis of variance or a student t-test where appropriate. Post hoc comparisons versus vehicle group were performed using Dunnett’s test. Differences among groups were considered statistically significant if *p* < 0.05.

## 3. Results

### 3.1. NVP-AEW541 Inhibited Growth and Led to Impaired Glucose Tolerance in Juvenile Rats

We administered NVP-AEW541 to juvenile rats in order to assess its effect on growth and development based on organ and body weights. Figure 1 shows the impact of 14 days of oral NVP-AEW541 application on the body and organ weights and tibia lengths of juvenile, growing rats. All dosages (80–240 mg/kg BW) of the NVP-AEW541 treatment led to a markedly reduced body weight (Figure 1A), as well as reduced weights of all evaluated organs (Figure 1B–D,F–H), suggesting that NVP-AEW541 has extensive effects on the whole body and all kinds of different tissues, i.e., fat, cardiac and skeletal muscle, the liver, and the lung. Since animals of this age are still growing, reduced organ weights and especially shorter tibia lengths may indicate the growth retardation of treated animals, reflecting the purpose of the use of NVP-AEW541 to inhibit the growth of tumor tissues. Remarkably, the reduction in the body and organ weights and tibia lengths showed a strong dose dependency. Juvenile rats showed unchanged fasting blood glucose levels during NVP-AEW541 treatment. However, blood glucose removal during the glucose tolerance test was delayed, indicating impaired systemic glucose tolerance. This effect, again, was dose dependent.

As proof of concept, these data demonstrate the bioavailability after oral administration and the expected effect of the NVP-AEW541 treatment on growth and development. Since the lowest dose of 80 mg/kg was effective, it was used for further assessment in adult rats.

### 3.2. NVPAEW-541 Impacts Body and Organ Weights and Glucose Tolerance in Adult Rats

**Figure 2:**
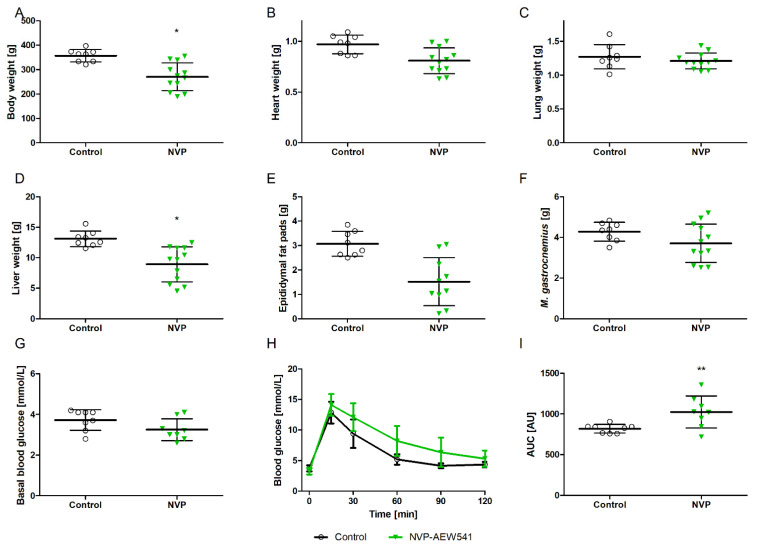
Effects of two weeks of NVP-AEW541 treatment (80 mg/kg) on the body and organ weights and the glucose tolerance of adult rats. Body weight (**A**), organ weights (**B**–**F**), fasting blood glucose (**G**), time course of glucose removal (**H**), and glucose tolerance (**I**) as determined by area under the curve (AUC). Data are presented as mean ± SD, *n* = 8–12 per group. Significant differences * *p* < 0.05; ** *p* < 0.01 vs. vehicle.

In addition to juvenile rats, we treated full grown adult male rats to assess whether NVP-AEW541 influences their body or organ weights. Figure 2 shows the impact of oral NVP-AEW541 application on the body and organ weights of the adult rats. NVP-AEW541 had no effect on the tibia length of the adult rats (38.4 vs. 37.5 mm; *p* = 0.2). NVP-AEW541 application for two weeks led to a reduction in body (Figure 2A) and organ weights (Figure 2B–F). Comparably to the juvenile rats, the adult rats showed unchanged fasting blood glucose levels (Figure 2G). However, blood glucose removal during the glucose tolerance test was, again, delayed (Figure 2I, AUC for glucose removal was significantly elevated), demonstrating disturbed glucose tolerance in the NVP-AEW541-treated adult rats.

### 3.3. Impact of NVP-AEW541 on Cardiac Morphology and Contractile Function

**Figure 3:**
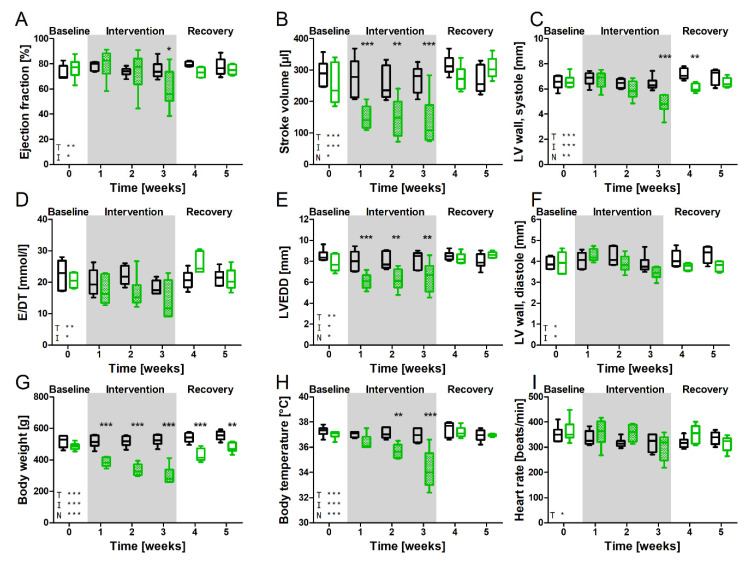
Parameters of left ventricular (LV) cardiac morphology, body weight, body temperature, and heart rate at baseline, throughout NVP-AEW541/vehicle treatment, and during recovery. Ejection fraction (**A**), stroke volume (**B**), systolic (**C**) and diastolic (**F**) LV wall thickness, time velocity index of E-wave (E/DT) (**D**), LV end-diastolic diameter (LVEDD) (**E**), body weight (**G**), body temperature (**H**) and heart rate (**I**). Black boxes indicate controls, *n* = 6; green boxes indicate NVP-AEW541-treated animals, *n* = 6. Data are mean ± min/max. Significant differences between treated and control animals (2-way ANOVA analysis), * *p* < 0.05; ** *p* < 0.01; *** *p* < 0.001. N indicates overall differences of NVP-AEW541, T indicates overall differences over time, and I indicates differences based on interaction between treatment and time. NVP-AEW541 affected cardiac protein expression and phosphorylation of Ins/IGF-1R and Akt.

There is a lack of information on NVP-AEW541 effects, especially on the heart. Thus, we investigated cardiac function and morphology echocardiographically during the 3 weeks of treatment and subsequent recovery. Figure 3 shows the echocardiographic parameters, body weight, body temperature, and heart rate of the rats before treatment; after one, two, and three weeks of NVP-AEW541 application; and for two weeks following the cessation of treatment. NVP-AEW541 treatment led to impaired contractile function with reduced ejection fraction (Figure 3A) after three weeks of treatment (*p* = 0.05). Cardiac stroke volume was decreased at every time point of NVP-AEW541 treatment (Figure 3B), indicating the impact of NVP-AEW541 application on cardiac contractile function (overall impact of NVP-AEW541 *p* = 0.01). Slightly reduced left ventricular end-diastolic dimension (LVEDD) (Figure 3E) (*p* = 0.02) suggests a mild impact on ventricle relaxation, although the diastolic parameter of E/DT (Figure 3D) was not affected. Left ventricular wall (LV wall) thickness was unchanged in diastole (Figure 3C) but decreased in systole (Figure 3F) (*p* = 0.8 and *p* = 0.01), which is consistent with atrophy, as observed in other organs (Figure 2) and the reduction in heart weight. Heart rates were comparable between NVP-AEW541-treated and control animals. Three weeks of treatment resulted in a profoundly reduced body weight (*p* = 0.001) and a lower body temperature (*p* = 0.0005) in a time-dependent manner (*p* < 0.0001). Continuing treatment seemed no longer tenable. Thus, we decided to terminate the chronic application of NVP-AEW541. Within the next two weeks, body weights and temperature recovered substantially, and cardiac function nearly normalized. Ejection fraction, stroke volume, and ventricular dimensions recovered completely.

**Figure 4:**
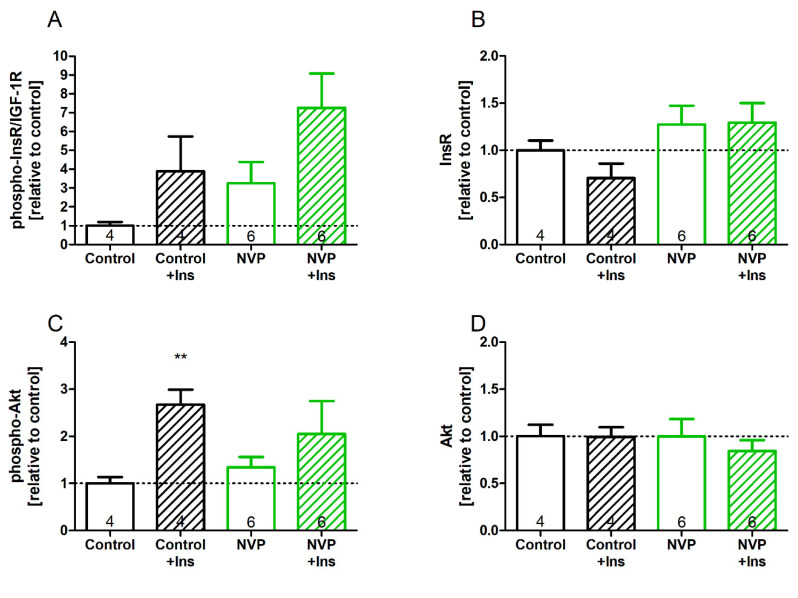
Cardiac insulin signaling after two weeks of NVP-AEW541 treatment: Phosphorylation of InsR/IGF-1R (**A**) and downstream signaling protein Akt (**C**), as well as total protein expression of insulin receptor (InsR) (**B**) and Akt (**D**) with and without insulin stimulation (+Ins). Data are mean ± SEM, *n* = 4–6 per group as indicated in the bars. Significant differences ** *p* < 0.01 vs. vehicle.

The reduced systemic glucose tolerance and treatment effects on the heart led us to analyze cardiac insulin signaling at the level of the protein expression and phosphorylation of InsR/IGF-1R. Figure 4 shows the protein expressions of cardiac insulin (InsR)/IGF-1 receptors (4A), as well as their phosphorylation ratios (4B), after two weeks of chronic oral NVP-AEW541 treatment. The levels of phosphorylated InsR/IGF-1R were comparable in the NVP-AEW541-treated animals and controls (*p* = 0.15) (Figure 4A). Although not reaching statistical significance, insulin stimulation showed a tendency to increase the phosphorylation of InsR/IGF 1R in both the NVP-AEW541 and control animals (*p* = 0.09 vs. *p* = 0.17). Interestingly, the basal expression of InsR/IGF-1 (Figure 4B) was slightly elevated in the hearts of the NVP-AEW541-treated rats compared to that in the hearts of the controls (*p* = 0.056), indicating an increase in total receptor expression. This may be a possible compensatory mechanism used to account for lower phosphorylation (activation) levels upon stimulation of the receptors by its ligand insulin. We additionally analyzed the phosphorylation (Figure 4C) and expression (Figure 4D) levels of Akt (protein kinase B). Akt is a downstream signal of both insulin receptor and IGF receptor stimulation. The phosphorylation of Akt upon insulin stimulation (Figure 4C) was increased in the controls (*p* = 0.003), indicating normal InsR/IGF-1R downstream signaling. The hearts of the NVP-AEW541-treated animals showed no significant increase in the phosphorylation of Akt (*p* = 0.356), suggesting less effective signaling of Ins/IGF-1R. The total protein expression of Akt (Figure 4D) was not different between the treated and control hearts.

### 3.4. NVPAEW Reduced Insulin Response but Did Not Affect Cardiac Function in Isolated Working Hearts

**Figure 5:**
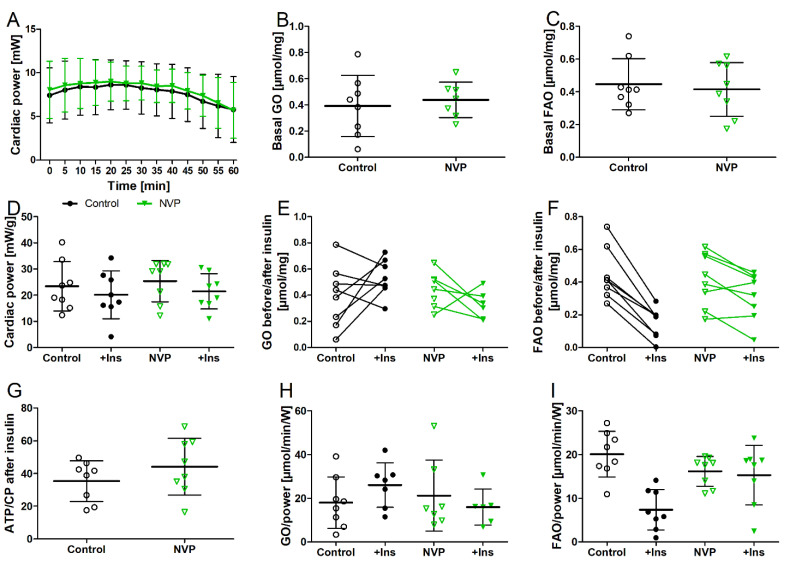
Effects of acute NVP-AEW541 (NVP) application on isolated working hearts. Cardiac power (**A**,**D**), basal glucose and fatty acid oxidation(**B**,**C**), insulin response (+Ins) of glucose oxidation (GO) and fatty acid oxidation (FAO) (**E**,**F**,**H**,**I**), and calculated ATP turnover per cardiac power (**G**). Data are presented as mean ± SD, *n* = 8 per group.

We used isolated working hearts, which allows for the direct measurements of acute changes in cardiac function and substrate metabolism, as well as insulin response. Such data are not obtainable in vivo. Figure 5 shows the effects of acute NVP-AEW541 application in the isolated working hearts on cardiac function and substrate metabolism. In contrast to oral treatment in vivo, the acute application of NVP-AEW541 to the isolated hearts did not reduce cardiac power (Figure 5A). The baseline rates of glucose (GO) and fatty acid oxidation (FAO) were comparable between the treated and control hearts (Figure 5B,C). The addition of insulin did not affect glucose oxidation (*p* = 0.2) (Figure 5E) but significantly reduced fatty acid oxidation rates (*p* < 0.001) (Figure 5F) in the control hearts. Of note, in the hearts treated with NVP-AEW541, fatty acid oxidation only mildly decreased (*p* = 0.02). We related fatty acid oxidation, glucose oxidation, and ATP production to cardiac power (Figure 5G–I). This calculation allows for a better comparison of hearts with different levels of cardiac power. Furthermore, the ATP-to-power ratio gives an estimate of how much ATP is needed for a certain amount of cardiac work. Relating fatty acid oxidation to cardiac power (Figure 5I) showed insulin-induced reductions in the control hearts (*p* = 0.0005) as expected but not in the NVP-AEW541-treated hearts (*p* = 0.77). In addition, the calculated production of ATP per power produced (Figure 5G) was higher in the NVP-AEW541-treated hearts than in the control hearts. This suggests reduced cardiac efficacy in the NVP-AEW541-treated hearts.

Table 1 shows the further effects of NVP-AEW541 on the cardiac function of the isolated working hearts. The treated hearts had lower heart rates, which was associated with increased values of developed pressure (dP/dt) and higher stroke volumes such that cardiac power remained the same.

## 4. Discussion

We demonstrated here that the systemic administration of NVP-AEW541 led to a dose- and time-dependent impairment of glucose tolerance, growth, and cardiac function. Since cardiac insulin signaling was maintained in vivo but impaired in vitro, and contractile function was not affected in vitro; a direct link between insulin resistance and contractile dysfunction appears unlikely.

NVP-AEW541 treatment led to an impairment in cardiac function in adult rats. These changes became more pronounced with an increasing length of treatment. After three weeks of treatment, systolic functional changes became so severe that continued application seemed to be no longer compatible with survival. However, both the impairment in systolic function and the reduced diastolic function were found to be fully reversible after treatment was terminated. It has been described that IGF-1 regulates cardiac contractility and output [13,14] and that it maintains the heart’s normal function and homeostasis [8,9]. Furthermore, low levels of IGF-1 are related to congestive heart failure [15], and receptor deficiency in cardiac muscle even leads to early death from heart failure in mice [16]. These findings are consistent with our results showing the depression of cardiac contractile function upon IGF-1R receptor inhibition with NVP-AEW541. However, the immediate recovery of cardiac contractility suggests the absence of a persisting structural impairment of the myocardium. The effects on cardiac function might instead be related to disturbances in cardiac homeostasis, which might not exclusively be maintained by cardiomyocytes but by a broad spectrum of different cells and signals [17,18].

In the present investigation, two weeks of oral treatment with NVP-AEW541 led to impaired growth with reduced body and organ weights in juvenile rats. The systemic effects of NVP-AEW541, as one representative of this treatment class, were dose dependent. Our selected dosages are comparable to those used in previous studies with rodents [2,4]. However, in previous investigations, there was no analyses of the body and organ weights of the juvenile animals included. In the adult rats, NVP-AEW541 treatment led to a similar reduction in body and organ weights. In two analyses [4,19], the authors described a loss of body weight. In contrast, further manuscripts found no effect of NVP-AEW541 on body or organ weight [2]. However, in the aforementioned study, this analysis was performed in adult mice receiving slightly lower doses, which may be a reason for the discrepancy described.

Our analysis of cardiac insulin/IGF signaling revealed reduced insulin/IGF-1 receptor phosphorylation in response to insulin stimulation. This may suggest insulin resistance. However, the phosphorylation of Akt as a central mediator in both pathways was not different with NVP-AEW541 treatment. Furthermore, the protein expression of the insulin/IGF receptors was increased. Both aspects indicate that insulin sensitivity may not be affected with chronic treatment or may be compensated for. Similar findings have been previously reported as an obstacle in the selective inhibition of IGF-1R as antitumor therapy [20]. Thus, the effect of long-term treatment with NVP-AEW541 may only be small.

NVP-AEW541 has been reported to not increase plasma glucose levels [2] or cause hyperglycemia [4]. In our investigation with two weeks of NVP-AEW541 treatment, we found no changes in plasma glucose levels in the juvenile or adult animals. Thus, our results are comparable to the earlier measurements. However, we additionally analyzed glucose tolerance using an intraperitoneal glucose tolerance test, which revealed dose-dependent impairment in glucose consumption in weanlings. In the adult animals, NVP-AEW541 treatment also led to impaired glucose tolerance, confirming the effect seen in the young rats. Our results indicate that NVP-AEW541 affects insulin signaling despite its reported more selective affinity to the IGF receptor. It may be expected that other substances of the treatment class have similar or even stronger effects depending on their receptor specificity. Thus, the plasma glucose level does not seem to be sufficiently sensitive for the assessment of the effects of NVP-AEW541 on insulin signaling and glucose metabolism.

We determined the acute effects of NVP-AEW541 on metabolism and cardiac function using isolated working rat hearts. Here, NVP-AEW541 did not affect cardiac function. Instead, NVP-AEW541 impaired the typical increase in glucose oxidation and the reduction of fatty acid consumption in response to insulin. We could not find any analysis assessing cardiac or muscular metabolism in response to IGF-1 inhibition. The inhibition of insulin signaling in the isolated rat hearts supports the reasoning that IGF-1R and the insulin receptor are highly homologous and interact both on insulin and on IGF-1 signaling [21,22]. This is also supported by reports describing heterologous reporters of IGF-1 and InsR [23]. NVP-AEW541 has been suggested to be more specific for the IGF-1 receptor [3], but our results suggest that the interaction of NVP-AEW541 with the insulin receptor or with the heterodimeric receptor may not be negligible.

Furthermore, our results with acute and chronic NVP-AEW541 treatment may be conflicting. While acute treatment is connected to insulin resistance with no functional impairment, chronic treatment comes with cardiac functional impairment but no effect on insulin sensitivity. From these data, we conclude that insulin resistance and contractile dysfunction do not seem to be directly connected. It may be speculated that non-contractile cellular signaling and adaption mechanisms mediate NVP-AEW541′s impact on cardiac function.

The systemic administration of NVP-AEW541 led to a dose- and time-dependent impairment of glucose tolerance and reduced growth and weight loss. We describe for the first time a severe impairment of cardiac contractile function with an increasing duration of treatment. The preservation of cardiac insulin signaling in vivo and impairment ex vivo, together with the absence of direct effects on cardiac function ex vivo, suggest that a direct link between insulin resistance and contractile dysfunction appears unlikely.

## Figures and Tables

**Table 1:** Effects NVP-AEW541 on cardiac function during ex vivo isolated heart perfusion.

Treatment	No Insulin		with Insulin	
	Control	NVP-AEW541	Control	NVP-AEW541
Heart rate (bpm)	167 ± 29	137 ± 29 *	182 ± 20	137 ± 19 *
Cardiac output (mL/min)	46.9 ± 14.8	54.2 ± 6.0	43.4 ± 20.0	47.5 ± 13.6
Stroke volume (µL)	282 ± 64	363 ± 130 *	254 ± 106	348 ± 98 *
dp/dt max (mmHg/s)	557 ± 128	711 ± 118 *	492 ± 165	663 ± 142 *

Data are mean ± SD. *n* = 8 per group. Significantly different * *p* < 0.05 vs. vehicle.

## Data Availability

The data presented in this study are available on request from the corresponding author. The data are not publicly available due to yet unpublished ongoing human studies involving the described treatment.

## Supplementary Materials

The following supporting information can be downloaded at: https://www.mdpi.com/article/10.3390/biomedicines10082022/s1.
