# Supplementary material for: The IGF-1R Inhibitor NVP-AEW541 Causes Insulin-Independent and Reversible Cardiac Contractile Dysfunction
Source: Biomedicines. 2022 Aug 19;10(8):2022. doi: 10.3390/biomedicines10082022 (PMC9406171; doi:10.3390/biomedicines10082022)
Supplement: Supplementary file 1 [file biomedicines-10-02022-s001.zip › biomedicines-1864172-supplementary.pdf]

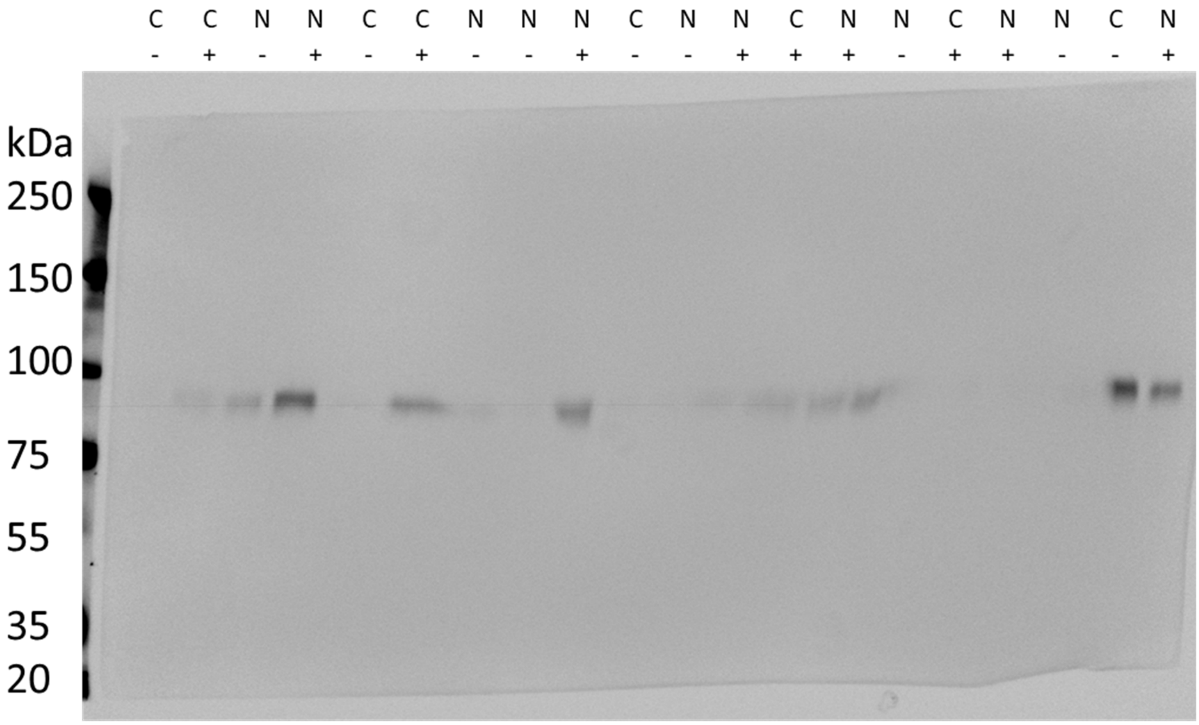

Figure S1 Western blot membrane, incubated with anti phospho-Insulin/IGF-1 receptor (95 kDa) of NVP-AEW541 (N) treated and control (C) animals stimulated with insulin (+) or without stimulation (-).

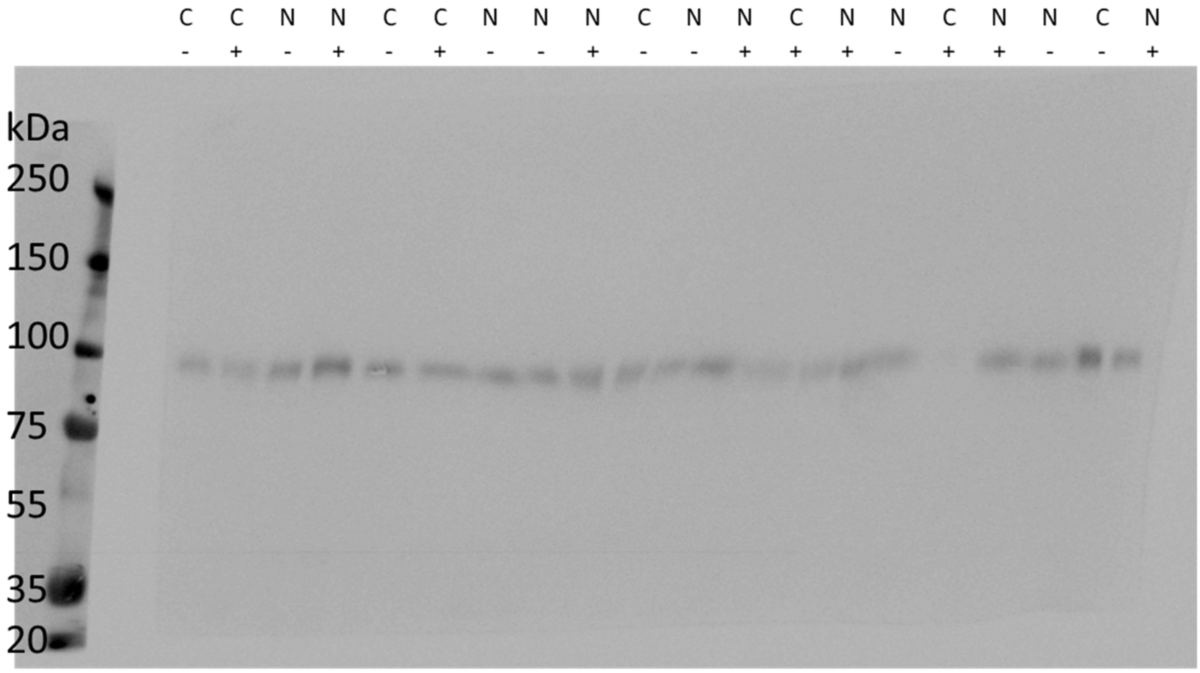

Figure S2 Western blot membrane, incubated with anti-Insulin receptor (95 kDa) of NVP-AEW541 (N) treated and control (C) animals stimulated with insulin (+) or without stimulation (-).

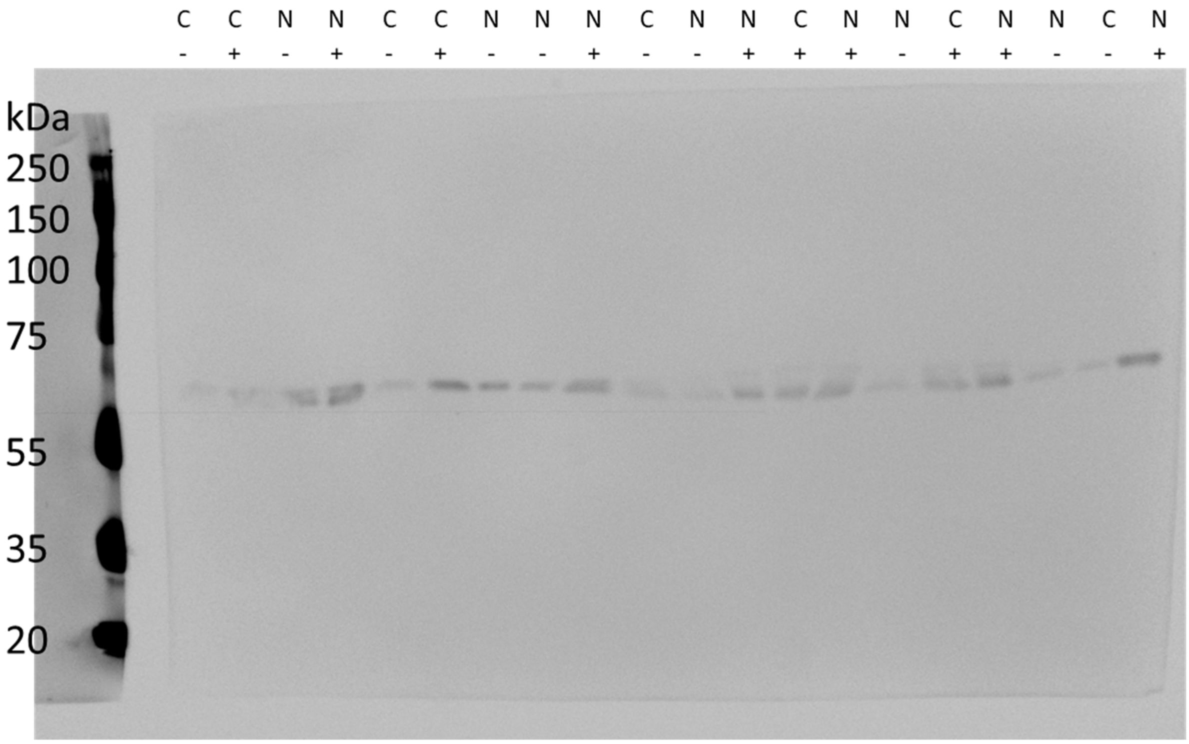

Figure S3 Western blot membrane, incubated with anti phospho-Akt (60 kDa) of NVP-AEW541(N) treated and control (C) animals stimulated with insulin (+) or without stimulation (-).

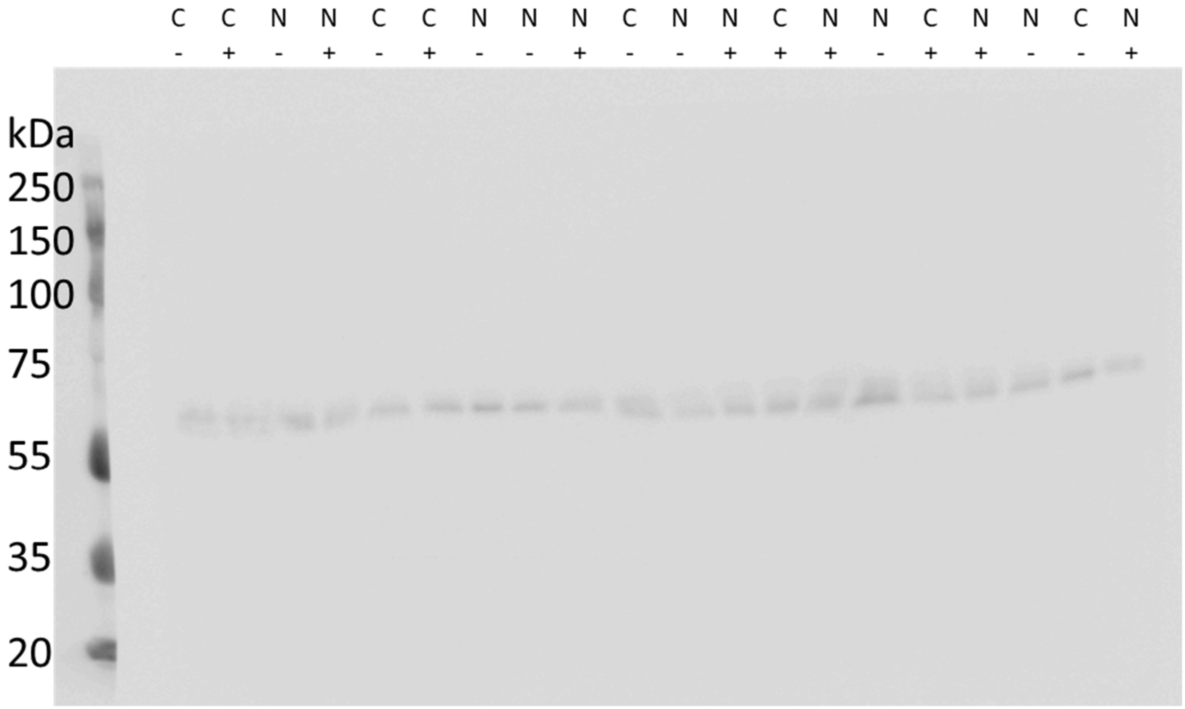

Figure S4 Western blot membrane, incubated with anti-Akt (60 kDa) of NVP-AEW541 (N) treated and control (C) animals stimulated with insulin (+) or without stimulation (-).
